# Two-year quality of life after free flap reconstruction in tumor-site discrepancy among Taiwanese with moderately advanced oral squamous cell carcinoma

**DOI:** 10.1186/1477-7819-10-145

**Published:** 2012-07-13

**Authors:** Kao-Ping Chang, Chung-Sheng Lai, Tung-Ying Hsieh, Yi-Chia Wu, Chih-Hau Chang

**Affiliations:** 1 Division of Plastic and Reconstructive Surgery, Department of Surgery, Kaohsiung Medical University Hospital, Kaohsiung, Taiwan; 2 Department of Surgery, Faculty of Medicine, College of Medicine, Kaohsiung Medical University, Kaohsiung, Taiwan; 3 Division of Plastic and Reconstructive Surgery, Kaohsiung Medical University Hospital, 100 Shih-Chuan 1st Road, Kaohsiung, 807, Taiwan

**Keywords:** Quality of life, T4a oral cancer, Free flap transfer, Questionnaire, Taiwanese

## Abstract

**Background:**

This study describes 2-year impact on quality of life (QOL) in relation to the anatomical discrepancy among T4a oral cancer patients after free flap reconstruction in Taiwan.

**Methods:**

Thirty-two patients who underwent tumor ablation with simultaneous microvascular free flap transfer at 2-year follow-up were recruited. They were divided into six subgroups, according to the resected area, consisting of: (1) buccal/retromolar trigone; (2) cheek; (3) commissure; (4) lip; (5) mandible; and (6) tongue. Functional disturbances and daily activity were analyzed using the Version-1 UW QOL Questionnaire with one more specific category: ‘Drooling’. Kruskal-Wallis rank sums analysis was used to test differences in average QOL scores between these subgroups. *Post-hoc* analysis was applied to assess influence of dominant categories between subgroups.

**Results:**

The category ‘Pain’ revealed the highest average score and reached significant statistical difference (*P* = 0.019) among all the categories, however, the category ‘Employment’ averaged the lowest score. Regarding ‘Pain’, there existed a statistical significance (*P* = 0.0032) between the commissure- and cheek-involved groups, which described the former showed poorer pain quality of life.

**Conclusions:**

The commissure-involved group had the lowest average score, which might imply the worst QOL in our study, especially for the categories ‘Pain’ and ‘Drooling’. This present study of T4a patients was the first carried out in Taiwan implementing the QOL questionnaire, and its results may serve for future reference.

## Background

In Taiwan, head and neck cancers present a major public health problem. Of these, oral squamous cell carcinoma ranks as the fourth most common malignancy among males according to the 2005 cancer registry [1], and betel nut chewing plays a critical role in the development of oral cancer. Patients with head and neck cancers not only have to face a life-threatening disease, but have to deal with the impact of the disease and the resulting surgical intervention on their quality of life (QOL) as well. Therefore, health-related QOL has been increasingly thought to be of paramount importance in the assessment of surgical results of oral squamous cell carcinoma, especially in advanced T4a diseases [2].

The popular application of microvascular free tissue transfer has facilitated larger head and neck tumor resection, but there has been no significant change in overall survival [3]. However, microvascular free tissue transfer seems to offer objective functional benefits [4,5]. It could be postulated that using microvascular free tissue reconstruction after resection may improve the QOL and daily function status in patients’ survival time. There have been few studies of longitudinal changes in QOL after free tissue reconstruction in patients with advanced head and neck cancer [6-9], but there are no studies concerning T4a patients with respect to individual anatomic location.

Although QOL is by definition a global concept, we would rather use the term ‘quality of life’ in a function-related manner, referring to the patient’s abilities to perform daily activities, such as eating and swallowing, after specific procedures [10]. However, studies that try to verify this result have been lacking, especially in Asia. The purpose of this investigation was, based on subjective questionnaire, to assess the impact on QOL of Taiwanese patients with T4a oral cancers in various anatomic locations after 2-year free flap reconstruction and to offer a guide for head and neck cancer surgeons to predict and explain the possible postoperative QOL to patients with different tumor locations.

## Methods

The study population was treated at the Department of Plastic and Reconstructive Surgery of Kaohsiung Medical University Hospital (KMUH), Kaohsiung, Taiwan. Patients were eligible for this study if they had advanced head and neck cancers and were to be treated by composite resection with immediate microvascular free tissue transfer. From January 2006 to November 2009, 32 patients met the additional criteria of histological findings (squamous cell carcinoma); restriction of their cancers to T4a disease; and a lapse of 2 years (range, 24 to 26 months) since reconstructive flap surgery. Furthermore, all patients recruited in our study received no flap revision after free flap reconstruction to eliminate remodeling bias. However, those patients who were deceased, lost to follow-up for more than 6 months at the time of investigation, or involved with more than one resected defect with more than one surgery were excluded from this study due to confounding complexity of functional analysis. Postoperative adjuvant radiotherapy was indicated for patients with free but close tumor margin <1 cm or involved lymph node metastases. These patients were divided into six subgroups, according to the resected area, consisting of: (1) buccal/retromolar trigone: involving the inner oral cavity but with adjacent structure invasion; (2) cheek: involving through-and-through full-thickness defect; (3) commissure: involving commissural defect; (4) lip: involving upper or lower lip without commissure defect; (5) mandible: involving segmental en-bloc bony resection; and (6) tongue: involving more than half of the tongue resection.

### Clinical examination

The questionnaire design was based on the University of Washington Quality-of-Life Head and Neck Questionnaire (UW-QOL, version 1) [11]. Our modification consisted of one more category: ‘Drooling’ (Table 1). Drooling is both debilitating and a marker of poor swallowing and lip function, and much more complained of postoperatively by Taiwanese patients. This questionnaire elicits responses from the patient and is entirely self-evaluated. The scale consists of 10 categories, each of which describes important daily living dysfunction or limitations. Within each category the highest level provides 100 points (best QOL) and each subsequent lower level provides an even reduction of points, whereas the lowest level or greatest dysfunction is only scored 0 point (worst QOL). That means the range of response is 100, 75, 50, 25, 0 for five options, 100, 67, 33, 0 for four options, and 100, 50, 0 for three options.

**Table 1:** KMUH Head and Neck 10-item Questionnaire (KMUH-QOL):UW-QOL Questionnaire Version 1 plus one more item ‘Drooling’

**Pain**	
I have no pain.	100
There is mild pain but I do not need medication.	75
I have moderate pain (requires regular medication, such as codeine or non-narcotic).	50
I have severe pain controlled only by narcotics.	25
I have severe pain not controlled by medication.	0
**Disfigurement**	
There is no change in my appearance.	100
The change in my appearance is minor.	75
My appearance bothers me but I remain active.	50
I feel significantly disfigured and limit my activities due to my appearance.	25
I cannot be with people due to my appearance.	0
**Activity**	
I am as active as I have ever been.	100
There are times when I can’t keep up my old pace, but not often.	75
I am often tired and have slowed down my activities although I still get out.	50
I don’t go out because I don’t have the strength.	25
I am usually in bed or a chair and don’t leave home.	0
**Recreation/Entertainment**	
There are no limitations to recreation at home and away from home.	100
There are a few things I can’t do but I still get out and enjoy life.	75
There are many times when I wish I could get out more but I’m not up to it.	50
There are severe limitations to what I can do. Mostly I stay home and watch TV.	25
I can’t do anything enjoyable.	0
**Employment**	
I work full time or job has no correlation with cancer.	100
I have a part-time but permanent job.	75
I only have occasional employment.	50
I am unemployed.	25
I am retired.	0
**Eating - Chewing**	
I can chew as well as ever.	100
I can eat soft solids but cannot chew some foods.	50
I cannot chew even soft solids.	0

The reason why we chose UW-QOL version 1 is that it is simple to answer for our patients, and category ‘Employment’ is much more concerning for Taiwanese patients. Although the addition of one domain (drooling) based on our empirical experience prevents it from retaining its originally well-established properties, our study primarily aimed to provide more information for patients with various tumor locations to understand possible postoperative QOL while preoperative consultations.

### Statistical analyses

For some reasons (for example, small sample sizes, lack of variability with data as some samples had SD = 0), Kruskal-Wallis rank sums analysis (non-parametric test) was used to test differences in QOL average scores between these different groups. *Post-hoc* analysis was applied to assess influence of dominant categories between subgroups of sampled populations. For all statistical tests, significance and borderline significance were defined as a *P* value ≤0.05, 0.1, irrespectively.

## Results

The 32 patients ranged in age from 34 to 68 years, with a mean age of 53.53 ± 7.96 years. All of them had T4a disease, and the majority were male (31, 96.88 %). Microvascular free tissue transfers included anterolateral thigh flap (20, 62.5 %), fibula flap (4, 12.5 %), both anterolateral thigh flap with fibula flap (2, 6.25 %) and radial forearm flap (6, 18.75 %). We grouped these patients into six groups: buccal-involved (*n* = 3, 9.4 %), cheek-involved (*n* = 9, 28.1 %), commissure-involved (*n* = 5, 15.6 %), lip-involved (*n* = 5, 15.6 %), mandible-involved (*n* = 7, 21.9 %), and tongue-involved (*n* = 3, 9.4 %).

### Category ‘Pain’ reached the highest average score and category ‘Employment’ averaged the lowest score among the entire categories (Table 2)

**Table 2:** Kruskal-Wallis rank sums analysis in QOL average scores between subgroups

**Subgroup**	**Total**	**Pain**	**Disfigurement**	**Activity**	**Recreation**	**Employment**	**Eating-Chewing**	**Eating-Swallowing**	**Speech**	**Shoulder disability**	**Drooling**
Buccal (*n* = 3)	786.33 ± 122.66	100.00 ± 0.00	75.00 ± 25.00	91.67 ± 14.43	91.67 ± 14.43	33.33 ± 57.74	66.67 ± 28.87	78.00 ± 19.05	100.00 ± 0.00	100.00 ± 0.00	50.00 ± 25.00
Cheek (*n* = 9)	767.89 ± 123.86	100.00 ± 0.00	61.11 ± 30.90	91.67 ± 17.68	83.33 ± 25.00	52.78 ± 45.83	61.11 ± 22.05	74.11 ± 27.87	74.22 ± 22.23	89.00 ± 16.50	80.56 ± 24.30
Commissure (*n* = 5)	595.20 ± 214.62	75.00 ± 17.68	40.00 ± 37.91	70.00 ± 32.60	65.00 ± 28.50	35.00 ± 28.50	50.00 ± 35.36	53.20 ± 30.02	86.80 ± 18.07	80.20 ± 18.07	40.00 ± 37.91
Lip (*n* = 5)	743.60 ± 118.70	95.00 ± 11.18	55.00 ± 32.60	90.00 ± 13.69	95.00 ± 11.18	25.00 ± 43.30	70.00 ± 27.39	86.80 ± 18.07	93.40 ± 14.76	73.40 ± 27.97	60.00 ± 28.50
Mandible (*n* = 7)	735.00 ± 157.57	89.29 ± 19.67	57.14 ± 31.34	82.14 ± 23.78	75.00 ± 35.36	50.00 ± 50.00	57.14 ± 18.90	81.14 ± 17.64	76.29 ± 25.23	81.14 ± 17.64	85.71 ± 19.67
Tongue (*n* = 3)	795.00 ± 81.41	100.00 ± 0.00	66.67 ± 14.43	100.00 ± 0.00	100.00 ± 0.00	50.00 ± 50.00	66.67 ± 28.87	89.00 ± 19.05	78.00 ± 19.05	78.00 ± 19.05	66.67 ± 14.43
Mean ± SD	734.19 ± 148.34	92.97 ± 14.53	57.81 ± 30.08	86.72 ± 21.05	82.81 ± 25.74	42.97 ± 43.18	60.94 ± 24.54	76.13 ± 24.34	82.41 ± 20.66	83.47 ± 18.83	67.97 ± 29.26
*P* value	0.417	0.019^a^	0.736	0.485	0.263	0.831	0.860	0.374	0.281	0.438	0.066^#^

^a^Statistical significance, *P* <0.05; #borderline significance, *P* <0.1 (the sample size was sufficient to detect the difference in category ‘Drooling’ among the six groups of patients with an alpha of 0.1 and 0.9 power).

A *post-hoc* analysis reached significance (*P* = 0.0032) only between cheek- and commissure-involved groups in item ‘Pain.’

In the category ‘Pain’, there were 25 patients presenting ‘I had no pain’ and the average score of 32 patients approximated up to 93. It meant that most patients could tolerate this symptom well 2 years postoperatively. On the contrary, 18 of 32 patients answered ‘I am unemployed’ or ‘I am retired’ with an average score of the category ‘Employment’ being 43 and it was the lowest average score among the entire categories after 2-year postoperative follow-up. All patients in this study had been employed before surgery and there was no statistical significance (*P* = 0.403) between patients <60 years (*n* = 25) and patients >60 years (*n* = 7) in the category ‘Employment’. However, owing to averaged lowest score, the impact of tumor ablation with free flap reconstruction on postoperative employment needed to be well explained at preoperative consultation.

### Category ‘Pain’ showed significance and category ‘Drooling’ revealed borderline significance among subgroups

In Table 2, only the category ‘Pain’ reached statistical significance (92.97 ± 14.53; *P* = 0.019 < 0.05) and the category ‘Drooling’ showed borderline significance (67.97 ± 29.26; *P* = 0.066 < 0.1). Furthermore, a *post-hoc* analysis reached significance (*P* = 0.0032 < 0.05) between cheek- and commissure-involved) groups in the category ‘Pain’ (100.00 ± 0.00, 75.00 ± 17.68), but not in other subgroups. It revealed that the commissure-involved group showed poorer pain quality of life than cheek-involved group.

### Patients in the commissure-involved group had the tendency to suffer from poorer quality of life

The commissure-involved patients showed the lowest average score for the categories ‘Pain’ and ‘Drooling’(Table 2). It meant that they complained of more pain or discomfort, even 2 years postoperatively. Similarly, the commissure-involved group also complained of postoperative drooling more than other groups. Although it did not demonstrate statistical significance in the total average score among these subgroups, the commissure-involved patients had the lowest average total score and tendency to poorer postoperative QOL after 2-year follow-up in comparison with other groups.

## Discussion

In recent decades, with the development of microsurgical free tissue reconstruction, it is nevertheless commonly agreed that such patients can be rehabilitated earlier thereby better readapting to their social environment. Therefore, many reports on the repair of head and neck defects by free flaps have claimed that these techniques contribute to a higher level of QOL. This study aims to assess whether QOL is associated with different anatomic subsites among T4a oral cancer patients after free flap reconstruction, rather than answer the question whether free tissue transfer is associated with differences in QOL scores in oral cancers. That is why the patients were restricted to T4a disease in order to eliminate biases of tumor size and disease severity.

Table 2 reveals that the category ‘Pain’ averaged the highest score; approximately up to 93 after 2 years, but the category ‘Employment’ achieved the lowest mean scores compared with the other categories. All patients in this study had been employed before surgery. This indicates that head and neck cancer victims, even 2 years after reconstruction, experience difficulty in finding satisfactory employment in Taiwan, perhaps due to poor acceptance by the public. This finding could be interpreted in two ways. One is that these patients think of themselves as cancer victims with less aggressive attempt to compete with other people. The other one is that the public and employers treat them unfairly due to their disfigured appearances.

As seen from our questionnaire in Table 2, only the categories ‘Pain’ and ‘Drooling’ achieved (borderline) significant differences among the entire categories. Furthermore, the commissure-involved patients averaged the lowest score in both aspects. It might be noted that free flap reconstruction for commissure defect, due to tumor ablation, could lead patients to more discomfort and functional impairments. To construct a flap for reconstruction of the commissure through-and-through defect, two skin paddles for the inner and outer linings are needed. However, because the continuity of the sphincter muscle and the characteristic structure of the commissure cannot be rebuilt solely by the skin flap [12], it is hard to restore sphincter function and the shape of the lip involving the commissure and discomfort caused by involuntary movement on the flap could bother patients so much. A secondary revision of the flap is often needed for better function and cosmesis [13]. For simpler comparison, all patients recruited in our study did not receive flap revision after previous flap reconstruction. In addition, the commissure-involved group had the lowest average total score, as seen in Table 2. The results are reasonably explained in the following manner. The first is that patients with commissure involvement had a higher incidence of postoperative drooling. The second reason is that these patients had more conspicuous disfigurement in the perioral area [13], which might cause patients to be reluctant to maintain active interpersonal relationships after surgery.

Patients with head and neck cancers are prone to psychosocial changes because interpersonal relationships and intrapersonal expression depend largely upon the structural and functional integrity of the head and neck regions [14]. The impact of surgical parameters on the functional outcome of free flap reconstruction is complex because mutual relationships of individual parameters, such as location and size of the defect and reconstruction options, have to be taken into consideration. The descriptive data analysis of our study population showed that there existed differences in the categories ‘Pain’ (significant difference) and ‘Drooling’ (borderline significant difference) among the various subgroups. Although free tissue transfer could be used for resurfacing the defect after tumor ablation, we found that the anatomic subsite of the tumor location influenced QOL of cancer patients postoperatively. Therefore, prior to such surgeries, QOL after free tissue reconstruction should be thoroughly explained to patients with advanced head and neck cancers.

Disability resulting from co-morbid illness may influence patient perception of QOL; however, advanced co-morbidity is mainly associated with poorer survival [15,16]. Datema *et al*. reported that co-morbidity impacts overall survival and short-term mortality of the newly diagnosed patient with head and neck squamous cell cancer [17]. Therefore, co-morbidity seems to have a closer correlation with overall survival, rather than QOL. Furthermore, Gourin *et al*. [18] concluded that co-morbidity alone did not appear to affect 1-year post-treatment head and neck disease-specific QOL indices in their study and Ronis *et al*. [19] also reported that head and neck disease-specific domains were less affected by co-morbid illness. Therefore, co-morbidity could not be an independent category in our study.

However, there were still some limitations in this study. These comparisons might be somewhat flawed because of concerns over confounding primarily by T-stage and the small numbers and minor by multiple flap types and sizes, heterogeneous patient population, other adjunctive treatments (radiation, and so on) and some other subtypes of oral cancers, such as palate, larynx and hypopharynx (not only buccal, commissure, lip, and so on). These obstacles might be overcome in our future researches in this field.

## Conclusions

The impact of surgical parameters on the functional outcome of free flap reconstruction is complex because the anatomic subsite of tumor location influences the QOL of cancer patients postoperatively. Therefore, prior to such surgeries, future QOL after free tissue reconstruction should be thoroughly explained to patients for healthy doctor-patient communication and relationship. In brief, we provide a guide for head and neck cancer surgeons to predict the possible postoperative QOL after wide excision and immediate flap reconstruction.

## Consent

This study was reviewed and approved by the Human Investigation Review Committee at the Kaohsiung Medical University Hospital.

## Abbreviations

QOL, Quality of life; UW-QOL, University of Washington Quality-of-Life Head and Neck Questionnaire.

## Competing interests

The authors declare that they have no competing interests. There is no external source of funding involved in the submitted article.

## Authors’ contributions

K-PC drafted the manuscript, conceived the study, and carried out the literature research; C-SL conceived the study and realized the technique; T-YH carried out the literature review and helped in management of the patients; Y-CW helped in the preparation of the manuscript and data collection, analyses, and interpretation; C-HC carried out the literature review. All authors read and approved the final manuscript.
